# Gut hormone stimulation as a therapeutic approach in oral peptide delivery

**DOI:** 10.1016/j.jconrel.2024.07.007

**Published:** 2024-09

**Authors:** Ana Beloqui

**Affiliations:** a UCLouvain, Université catholique de Louvain, Louvain Drug Research Institute, Advanced Drug Delivery and Biomaterials, 1200 Brussels, Belgium; b WEL Research Institute, Avenue Pasteur, 6, 1300 Wavre, Belgium

**Keywords:** Gut hormone, Hormone stimulation, Oral peptide delivery, L cells, GLP-1, GLP-2

## Abstract

In this contribution to the Orations - New Horizons of the Journal of Controlled Release, I discuss the research that we have conducted on gut hormone stimulation as a therapeutic strategy in oral peptide delivery. One of the greatest challenges in oral drug delivery involves the development of new drug delivery systems that enable the absorption of therapeutic peptides into the systemic circulation at therapeutically relevant concentrations. This scenario is especially challenging in the treatment of chronic diseases (such as type 2 diabetes mellitus), wherein daily injections are often needed. However, for certain peptides, there may be an alternative in drug delivery to meet the need for increased peptide bioavailability; this is the case for gut hormone mimetics (including glucagon-like peptide (GLP)-1 or GLP-2). One plausible alternative for improved oral delivery of these peptides is the co-stimulation of the endogenous secretion of the hormone to reach therapeutic levels of the peptide. This oration will be focused on studies conducted on the stimulation of gut hormones secreted from enteroendocrine L cells in the treatment of gastrointestinal disorders, including a critical discussion of the limitations and future perspectives of implementing this approach in the clinical setting.

## Graphical abstract

**Figure:**
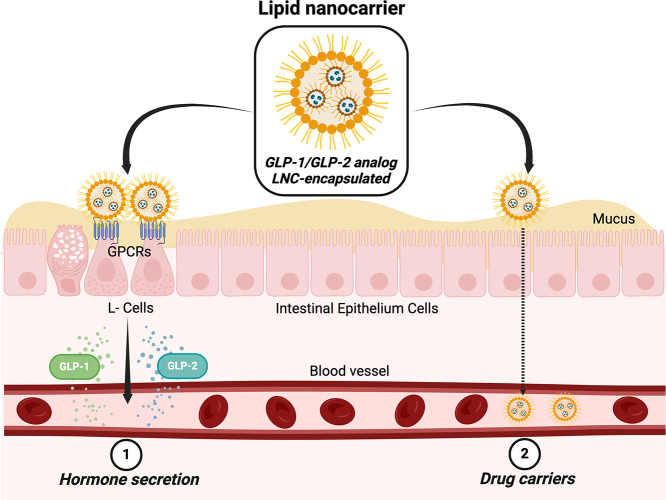

## 1 Introduction

The enteroendocrine system is the largest endocrine system in the body and secretes >20 different hormones involved in the treatment of different diseases [1]. Among the different types of enteroendocrine cells, L cells have attracted particular interest because of the pleiotropic effects of their secreted peptides (such as glucagon-like peptide (GLP)-1 and GLP-2) [2]. GLP-1 secreted from intestinal L cells stimulates postprandial insulin secretion in a glucose-dependent manner and is hydrolyzed within a few minutes by the enzyme dipeptidyl peptidase-IV (DPP-IV). Several DPP-IV inhibitors and GLP-1 analogs (with improved plasma half-lives, such as liraglutide and semaglutide) have been developed and proven to be highly successful in the treatment of type 2 diabetes mellitus (T2DM) and obesity [3]. Currently, there is only one oral formulation marketed for the delivery of the GLP-1 analog semaglutide (Rybelsus®, Novo Nordisk). It is based on the co-administration of the peptide with the permeation enhancer sodium N-[8-(2-hydroxybenzoyl) amino] caprylate (SNAC) and has a low bioavailability of ∼1%. The development of improved alternative drug delivery systems is of utmost importance to fulfill the potential of the oral route for the administration of these peptides.

The role of co-secreted GLP-2 is to help maintain physiological gut barrier function and regulate the stimulation of intestinal epithelial cell proliferation [4]. Teduglutide (Gattex®, Revestide®) has been commercialized as a daily subcutaneous injection for its use in short bowel syndrome (SBS), and glepaglutide is currently being evaluated in clinical trials for its use in SBS (clinicaltrials.gov identifier NCT03905707). Due to their intestinal growth-promoting activity, GLP-2 analogs hold promise as mucosal healing candidates for inflammatory bowel disease (IBD) treatment.

**Fig. 1:**
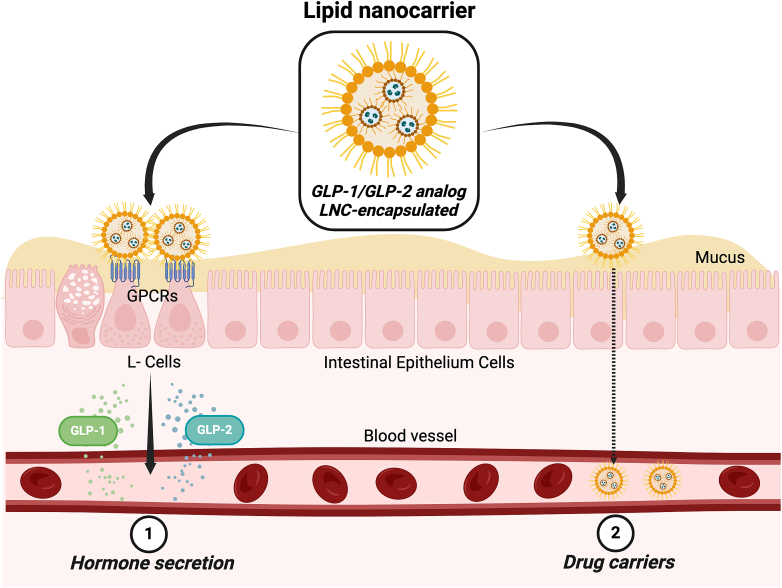
Schematic representation of the dual-action strategy *via* lipid nanocarriers for oral GLP-1/GLP-2 delivery. Created with Biorender.com.

An exciting alternative to peptide delivery would be to stimulate the secretion of the endogenous gut hormone. Few studies have demonstrated that using nutritional approaches may help to stimulate the endogenous release of incretin hormones in subjects with T2DM, thus providing sufficient stimuli to improve glycaemia [[5], [6], [7]]. When considering the clinical effectiveness of GLP-1 analogs and DPP-IV inhibitors, this represents an exciting new therapeutic approach that has yet to be exploited as a drug delivery strategy. L cells express different G protein-coupled receptors (GPCRs) that are activated by a wide variety of endogenous ligands found in the gut lumen [2]. Potential ligands for these receptors are lipids produced by the gut microbiota, such as short-chain fatty acids (SCFAs) (ligands for the receptors GPR43, GPR41 and GPR109a), medium-chain fatty acids (MCFAs) (GPR84) and long-chain fatty acids (LCFAs) (GPR40 and GPR120) [[8], [9], [10], [11]]. Although dietary interventions and nutrient manipulations (including changes in lipids) regulate the endogenous release of gastrointestinal hormones [12,13], nutritional strategies alone are usually not sufficient to fulfill the clinical needs of most patients with obesity and diabetes. However, drug delivery systems could be potential successful alternatives. Over the last decade, my group and I have been working on the hypothesis that we could stimulate the secretion of gut hormones while simultaneously providing plasma levels of the encapsulated peptide to overcome the previously mentioned limitation. In the present oration, I focus my attention on the oral delivery of GLP-1 and GLP-2 analogs by co-stimulating their endogenous secretion by enteroendocrine L cells *via* lipid-based formulations (schematic representation of the strategy is shown in Fig. 1).

## 2 Dual-action drug delivery systems for increased peptide concentrations

Considering that increasing endogenous GLP-1 secretion alone may not be sufficient to induce a therapeutic effect in the pathological context, we opted to develop a dual-action approach that combined the biological effect of lipid-based nanocarriers (inducing GLP-1 secretion) with that of the encapsulated molecule (a GLP-1 analog). As L cells express GPCRs on their surface that are activated by lipids, among other molecules, we hypothesized that lipid-based nanocarriers could mimic endogenous lipid ligands and induce GLP-1 secretion. First, we identified lipid carriers that would exert a stimulatory effect *per se* (including liposomes and lipid nanocapsules), without any encapsulated peptide, on gut hormone secretion. In our first study, we evaluated the effects of different lipid nanocarriers on L cell stimulation *in vitro* in murine and human L cells [14]. Among the investigated formulations, at the evaluated doses, nanostructured lipid carriers seemed to be the most promising at stimulating GLP-1 from L cells. However, *in vivo,* we did not observe any glucose-lowering effect with these nanocarriers, even when they were loaded with the GLP-1 analogs exenatide or liraglutide [15]. *Ex vivo* studies showed that these nanocarriers were mainly adhered to the mucus layer; hence, they did not reach the epithelium [15].

We then focused on lipid-based nanocapsules (LNC) because they are easy to produce and we can modify their lipid content and physicochemical properties, thus allowing us to study critical parameters that may interfere with the ability of the nanocarrier to stimulate gut hormone secretion [16]. The nanocapsules were made of Labrafac WL 1349 (medium-chain triglycerides), Solutol HS15 (polyethylene glycol (15)-hydroxystearate) and Lipoid S®100 (phosphatidylcholine). An increase in the lipid nanocapsule size and corresponding increase in the lipid content of the nanocapsules led to increased GLP-1 secretion from L cells *in vitro*. Regarding the *in vivo* condition, nanocapsules 200 nm in size were able to significantly increase GLP-1 levels in normoglycemic mice (4-fold greater than those in untreated mice at 1 h postadministration) [16].

Our hypothesis was that we could combine the increased endogenous GLP-1 levels induced by the nanocarrier with the plasma levels of the encapsulated peptide. We incorporated Peceol® (glyceryl monooleate) within the nanocapsules to decrease the temperature of the preparation procedure. Then, we encapsulated the GLP-1 analog exenatide within LNC by first encapsulating the peptide within reverse micelles (Span 80: Labrafac WL 1349 at a 1:5 wt ratio), thus obtaining an encapsulation efficiency of ∼85%. We conducted a pharmacokinetic study in normoglycemic and in obese/diabetic mice in a high-fat diet (HFD)-induced type 2 diabetes mellitus (T2DM) model (8 weeks of HFD treatment). Exenatide blood levels were significantly greater when lipid nanocapsules were orally administered than when exenatide was orally administered in solution to obese/diabetic mice (relative bioavailability of 4.32% with lipid nanocapsules, ***p < 0.001) [17] (Fig. 1F). Orally delivered peptides have advanced to the late phase of development, with an oral bioavailability between 0.5 and 1.0% [18]. This is also the case for Rybelsus® (Novo Nordisk®), which is the only orally administered GLP-1 analog that has been commercialized. However, exenatide plasma levels obtained with the nanocapsules in the T2DM model were not superior to those of previously described drug delivery systems for oral exenatide delivery [19,20].

## 3 Co-stimulation of GLP-1 leads to therapeutically relevant levels of the peptide in T2DM treatment

**Fig. 2:**
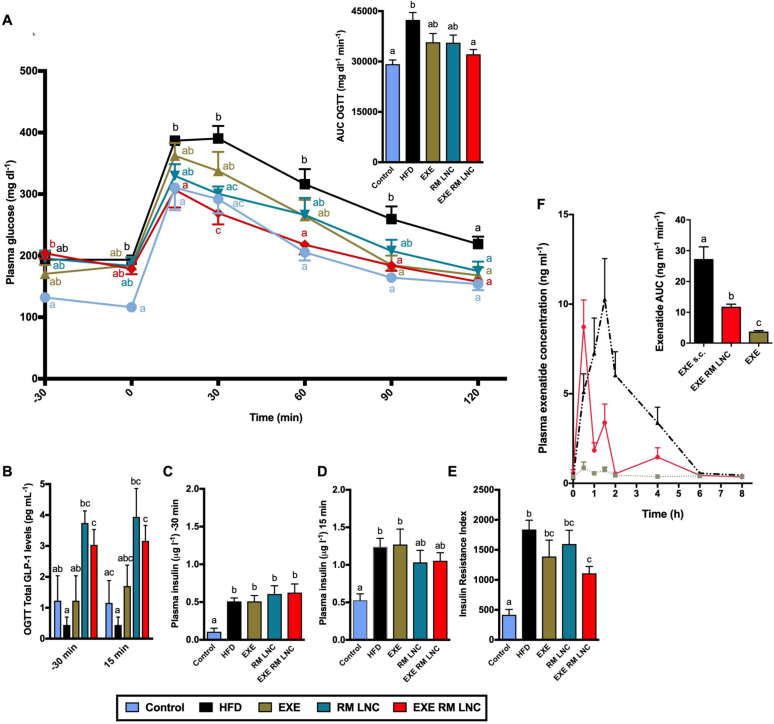
OGTT and pharmacokinetic evaluation of exenatide-loaded LNC in an HFD-induced obese/diabetic mouse model. (A) OGTT and mean area under the curve (AUC) (*n* = 8–9). (B) Plasma total GLP-1 levels and (C, D) plasma insulin levels measured 30 min before and 15 min after glucose challenge (n = 8–10). (E) Insulin resistance index (n = 8–9). (F) Concentration-time profile and AUC of exenatide after subcutaneous (EXE s.c.) (50 μg/kg exenatide dose) and oral administration of exenatide in solution and within RM LNC (EXE and EXE RM LNC, respectively) (at a dose of 500 μg/kg exenatide). The data are presented as the mean ± SEM (n = 8–10). The data with different superscript letters (A, B) are significantly different (*p < 0.05) according to two-way ANOVA followed by Tukey's *post hoc* test or (C—F) according to one-way ANOVA followed by Tukey's *post hoc* test. EXE: exenatide; RM LNC: reverse micelle LNC (unloaded); EXE RM LNC: exenatide-loaded reverse micelle LNC. Reprinted from [17]. Copyright® 2020 BMJ Publishing Group Ltd. & British Society of Gastroenterology.

The key question toward the exploitation of gut hormone stimulation as a therapeutic strategy in the management of chronic disorders is whether we can obtain therapeutically relevant levels of the peptide. To prove the efficacy of our strategy, we first evaluated the efficacy of our dual-action drug delivery system in the context of T2DM treatment using exenatide as a model GLP-1 analog [17]. GLP-1 is an incretin hormone that induces insulin secretion in a glucose-dependent manner. The incretin concept is based on the observation that oral glucose administration provides a more potent insulinotropic stimulus than intravenous glucose infusion [21]. Hence, we first tested the efficacy of our dual-action strategy following an oral glucose tolerance test (OGTT). We administered a 500 μg/kg exenatide dose (free and encapsulated within lipid nanocapsules (EXE RM LNC)) and equivalent concentrations of the nanocapsules or water 60 min before the oral glucose challenge (2 g/kg) (Fig. 2A). We chose this time point based on the increased secreted GLP-1 levels that were measured in our previous study in normoglycemic mice [16]. We observed that exenatide-loaded LNC (EXE RM LNC) completely normalized the glycemia, whereby they exhibited the same glucose profile as that observed in lean normoglycemic control mice (Fig. 2A), thus significantly decreasing plasma glucose levels and the glucose area under the curve (AUC). This was not the case for mice treated with exenatide alone. Total GLP-1 levels were significantly greater in both empty (RM LNC) and exenatide-loaded LNC (EXE RM LNC)-treated groups than in the control groups (control and HFD), thus confirming the ability of the nanosystem to stimulate GLP-1 release under pathological conditions (Fig. 2B). Unloaded nanocapsules alone lowered blood glucose levels compared with those in untreated HFD-fed mice. However, as we anticipated, this effect was not sufficient to reduce the hyperglycemia.

In the second step, we evaluated the effect of our strategy in the management of T2DM upon long-term treatment [17]. We administered different exenatide formulations daily (5 weeks) to obese/diabetic mice following the same administration doses as those used for the OGTT (500 μg/kg exenatide dose, free or encapsulated, and equivalent doses of unloaded nanocapsules). We included two subcutaneous exenatide injections (10 μg/kg exenatide dose): exenatide in solution (the same solution used for the preparation of exenatide-loaded LNC) or Byetta™, which is the marketed daily injection of exenatide. After 5 weeks of treatment, only the mice treated with exenatide-loaded LNC exhibited significantly decreased plasma glucose levels comparable to those in the nondiabetic control group (*p* > 0.05) and decreased insulin levels compared to those in the subcutaneous injection group. Insulin resistance was normalized in exenatide-loaded LNC-treated mice and was therapeutically equivalent to that of the marketed formulation (Byetta®). Overall, we observed that combining nanocarriers with GLP-1 analogues was sufficient to normalize the glycemia of obese/diabetic mice after either acute or chronic treatment.

Considering a foreseen translation into the clinical setting, we continued developing strategies to strengthen the secretory effect of the nanocarrier and/or prolong its antidiabetic effect *in vivo*. We developed fatty acid-targeted lipid nanocapsules to achieve this goal [22]. We did not observe any benefit *in vivo* when targeting the nanocarriers with fatty acids. However, nanocarriers that were surface-modified with DSPE-PEG_2000_ alone exhibited increased GLP-1 levels compared to unmodified nanocarriers, and this treatment increased GLP-1 levels up to 8-fold *in vivo* in normoglycemic mice. To test the therapeutic relevance of these increased GLP-1 levels, we conducted the same long-term study as previously described but followed two different administration regimens: either once daily or once every other day. Interestingly, compared with non-PEGylated exenatide-loaded nanocarriers, unloaded PEGylated nanocapsules administered once daily were also able to obtain basal glucose levels and significantly reduce insulin resistance, thus confirming that the increase in GLP-1 secretion (∼8-fold) was sufficient for decreasing glucose levels. Moreover, this effect was prolonged over time, thus allowing us to reduce the administration frequency. Hence, the secretory effect of the nanocarriers is tunable and could be further potentiated.

## 4 Co-stimulation of GLP-1 leads to therapeutically relevant levels of the peptide in metabolic dysfunction-associated steatotic liver disease (MASLD) treatment

GLP-1 analogs are currently being investigated in several clinical trials for the treatment of MASLD (liraglutide in the LEAN Project: ClinicalTrials.gov number NCT01237119 or semaglutide in ClinicalTrials.gov number NCT02970942 and NCT04822181) [23,24]. In our studies in T2DM obese/diabetic mice, we observed a profound effect of our formulation on liver steatosis (such as decreased lipid accumulation and decreased liver weight) [17]. We hypothesized that our orally administered dual-action strategy could exert a stronger impact on MASLD-associated metabolic syndrome than the subcutaneous injection of the GLP-1 analog, thus preventing progression to more severe disease states. We evaluated the impact of our nanosystem on the progression of the disease in two different mouse models of early MASLD (without fibrosis) (*foz/foz* mice fed an HFD and a dietary model (C57BL/6J mice fed with a western diet plus fructose (WDF)) that were subjected to chronic treatment for one month *vs.* a subcutaneous injection of the analog alone [25]. As in our previous studies, we used exenatide as a model GLP-1 analog.

It should be highlighted that both animal models exhibited different phenotypes, with WFD exhibiting less weight and lower starting glucose levels than *foz/foz*. Our strategy promoted the normalization of glucose homeostasis and insulin resistance in both models, thus mitigating the progression of the disease. In the liver, diverging results were observed between the models, with the *foz/foz* mice exhibiting better outcomes. Overall, oral administration of the nanosystem was more efficient at preventing the progression of the disease to more severe states than was subcutaneous injection. However, we did not observe differences between exenatide-loaded or unloaded LNC.

**Fig. 3:**
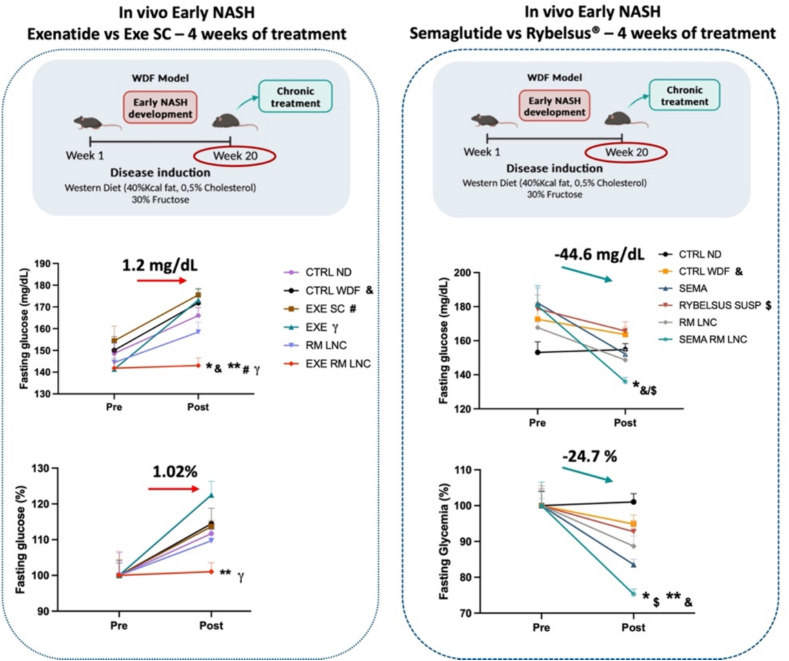
Glucose -lowering effect of exenatide- and semaglutide-loaded LNC in a WDF model of MASLD under fasting conditions. On the left, pre/post treatment fasting glucose (mg/dL and %) upon exenatide-LNC treatment compared to oral or subcutaneous exenatide. On the right, pre/post treatment fasting glucose (mg/dL and %) in the semaglutide-LNC treatment group compared to the oral semaglutide or Rybelsus® (in suspension) treatment groups. *P* values were determined *via* two-way ANOVA followed by Tukey's port hoc test. The data are represented as the mean ± SEM (*n* = 9–10). Adapted from [25] (Copyright® 2023 Elsevier B·V.) and [26] (Copyright® 2024 Springer Nature).

Considering the severity of the metabolic syndrome associated with MASLD (such as ∼400 mg/dL glucose basal levels in *foz/foz* mice *vs.* ∼200 mg/dL glucose basal levels in the HFD-fed mice), we hypothesized that we either needed to replace exenatide (half-life of ∼2.5 h) with a peptide with a longer half-life, such as liraglutide (∼13 h) or semaglutide (∼7 days), as per ongoing clinical trials, or prolong the treatment. We repeated the study in WFD-fed mice under the same conditions and followed the same administration regimen; however, we replaced exenatide with semaglutide and for this experiment, we did not compare the effect to that of subcutaneous injection but rather to that of Rybelsus® (semaglutide) (the oral GLP-1 marketed product) as a suspension (500 μg/kg exenatide and semaglutide dose) [26] (Fig. 3). We observed a better glucose-lowering effect with our formulation than with its subcutaneous (exenatide) or marketed (Rybelsus®) counterparts. These results also confirmed our hypothesis that replacing the GLP-1 analog exenatide with semaglutide could induce a more pronounced hypoglycemic effect.

The current trend in MASLD treatment involves the use of combination therapies targeting one or more “hits” (such as inflammation, oxidative stress and lipid accumulation) of disease development [27]. When considering the efficacy of our strategy at haltering the progression of MASLD, our approach demonstrates potential for combination therapies *via* the oral route, thus potentially leading to novel approaches for MASLD treatment.

## 5 Co-stimulation of GLP-2 leads to therapeutically relevant levels of the peptide in IBD treatment

Considering that GLP-2 is co-secreted with GLP-1, we hypothesized that we could exploit the secretory effect of lipid nanocapsules and develop an oral formulation for the delivery of the GLP-2 analog teduglutide [28]. We first confirmed the secretory effect of the nanocapsules *in vitro* in GLUTag murine L cells and *in vivo* in the context of the disease, thus demonstrating increased GLP-2 levels in both cases with unloaded lipid nanocapsules alone. We aimed to exploit the re-epithelizing effect of GLP-2 in the context of IBD treatment.

One of the main limitations in the use of GLP-2 in the context of IBD treatment in the clinical setting is the need to obtain therapeutic levels of the peptide in the chronic setting. Prolonged or increased doses of the peptide are needed regarding this scenario. GLP-2 is a re-epithelizing peptide and could thereby accelerate the intestinal growth of adenocarcinomas in IBD patients. To reduce a potential tumor-promoting effect, we and others have proposed short-term or intermittent use of GLP-2 or its analogs during the course of the disease [28,29]. We hypothesized that to potentiate the effect of GLP-2 and shorten the administration regimen, we could provide nanocapsules with anti-inflammatory properties while retaining the secretory effect of the nanocapsules. To do so, we functionalized our nanocarriers with the Lys-Pro-Val (KPV) tripeptide *via* hyaluronic acid (HA) conjugation, as this complex has been reported to be efficient in the context of IBD treatment [30]. Our strategy was to combine a re-epithelizing agent with an anti-inflammatory moiety within one nanoparticle.

We tested the efficacy of our KPV-HA-functionalized nanocapsules encapsulating teduglutide in a chronic murine IBD model. We subjected the mice to three cycles of dextran sodium sulfate (DSS) and opted for a 3-day administration regimen at the beginning of the “flare” episode (worsened state) of each DSS cycle, which started immediately after the removal of the DSS solution. We administered a total of 9 doses over 50 days. Treatment with our nanocapsules decreased the expression of most of the measured cytokines/chemokines while decreasing permeability (increased ZO-1 and Muc2). The secretion of GLP-2 by our nanocarriers induced a sufficient stimulation through mucosal healing upon intermittent administration of our formulation for 3 consecutive days at 3 times over 50 days, for a total of 9 doses. This strategy holds promise as a nanoparticle platform for combined mucosal healing and immunomodulation in IBD treatment.

**Fig. 4:**
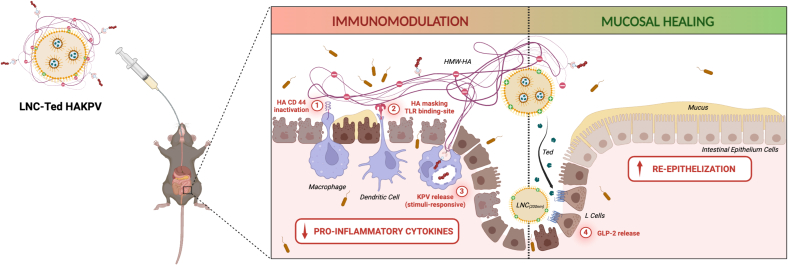
Schematic representation of the proposed mechanism of action of HA-KPV functionalized lipid nanocapsules (LNC-Ted HAKPV) in IBD treatment. The immunomodulation of HA-KPV is based on (1) the inactivation and (2) masking of immune cells from endogenous proinflammatory ligands associated with (3) the intracellular release of KPV. The mucosal healing effect is exerted by the lipid nanocapsules encapsulating teduglutide, which induces a local release of GLP-2. Reprinted from [28] (Copyright® 2023 Elsevier B.V).

A schematic representation of the proposed mechanism of action is depicted in Fig. 4.

## 6 Challenges, limitations and future perspectives

There are certain restrictions to the use of lipid-based drug delivery systems in the stimulation of gut hormones. The main limitation involves the high doses of lipids that may be needed in humans for a therapeutic effect if the dose-response extrapolated from mice to humans was linear. It should be highlighted that this is not the case for prebiotics that have been shown to stimulate endogenous GLP-1 secretion. Prebiotics are typically administered at a 10% dose in a rodent diet (200–300 mg/day) [31]; however, the dose that is administered to humans is not necessarily proportional. For example, in human clinical trials of prebiotics for GLP-1 stimulation, the dose was 16 g per day [32,33]. This would also need to be confirmed in the case of lipid nanocapsules. Not all lipid formulations stimulate gut hormone secretion *in vivo*, regardless of lipidicity. We first evaluated the use of nanostructured lipid carriers to achieve this goal; although the formulation performed very well *in vitro*, there was no effect *in vivo* [15]. This may be related to the fate of the nanocarriers *in vivo*, including the digestion of the formulation and/or diffusion across the mucus layer. *In vitro*, different lipid-based nanocarriers presented different abilities to stimulate L cells [14]. Further studies are needed to understand how these formulations sense enteroendocrine cells for gut hormone secretion and the physiological processes that trigger and/or contribute to this effect *in vivo*. This includes the evaluation of human specimens to evaluate whether the effect observed in mice could be translated to humans. Different lipid nanoparticle digestion products, such as fatty acids, may have an impact on the gut microbiota composition and hence an impact on the phenotype. It would be interesting to investigate whether the treatment with lipid nanocarriers could induce a shift in the gut microbiota composition in the pathological context, thus modulating specific groups of bacteria that are known to have an impact on the progression/regression of the disease. In addition, considering the foreseen commercialization of the formulation, the scale-up and manufacturing of nanocapsules for daily dosing in large populations (including T2DM patients, obese patients or IBD patients) need to be investigated.

Currently on the market, and in clinical trials, there are not only single GLP-1 receptor agonists available. Retatrutide, which is a triple agonist of the glucose-dependent insulinotropic polypeptide (GIP), GLP-1 and glucagon receptors, has completed phase II clinical trials for its use in obesity [34]. Survotide, glucagon/GLP-1 receptor dual agonist, has completed phase II clinical trials for its use in metabolic dysfunction-associated steatohepatitis (MASH) (clinicaltrials.gov identifier NCT04771273). Tirzepatide, which is a dual GIP and GLP-1 receptor agonist, has been approved for its use in the management of T2DM (Mounjaro®, Eli Lilly) and obesity (Zepbound®, Eli Lilly), both of which are injectable formulations. According to a large analysis of real-world data conducted by Truveta Research (not yet peer-reviewed) in overweight/obese adults in the US, tirzepatide appears to be more effective than semaglutide at reducing weight loss in this population [35]. GIP is also an incretin hormone; however, while GLP-1 suppresses glucagon secretion, GIP enhances postpandrial glucagon secretion. Both hormones are secreted from different cell types in different regions of the gut. GIP is secreted by K cells in the duodenum, whereas GLP-1 is secreted by L cells, which are scattered along the gastrointestinal tract at increasing concentrations as the tract transitions into the colon [36]. There is a different regional localization of these hormones. From a stimulatory perspective, if we were to stimulate the secretion of these or two or more other hormones, the challenge would be to modulate the secretion of the hormones targeting different regions of the gut simultaneously. Indeed, there are >20 gut hormones released from different enteroendocrine cells that act synergistically to tune different physiological processes both locally and peripherally. The selective targeting of one region of the gut is already a challenge; however, the simultaneous targeting of multiple regions is far more complicated. Recently, an ingestible electroceutical capsule for stimulation and modulation of ghrelin from the gastric mucosa has been developed [37]. However, further studies are needed to explore the stimulation capacity of these capsules in other regions of the gut. Ingestible devices may be useful for regional gut delivery; however, they will need to prove to be efficient at stimulating gut hormones in different gut regions and/or at multiple gut regions simultaneously in regard to simultaneous gut hormone modulation.

## 7 Conclusion

Gut hormone stimulation represents an exciting alternative to increased therapeutic peptide levels for oral peptide delivery. Dietary interventions regulate endogenous hormone secretion but are not sufficient for disease management. However, drug delivery systems could achieve this effect. In oral drug delivery, we could overcome the need to improve the limited oral bioavailability of oral peptide formulations if we could co-stimulate the secretion of endogenous hormones. There are different positive implications, which may include a potential reduction in the amount of peptide to be administered, the use of GRAS excipients and the use of the oral route to achieve this effect, thus avoiding the need for daily injections.

## CRediT authorship contribution statement

**Ana Beloqui:** Writing – review & editing, Writing – original draft, Validation, Supervision, Resources, Investigation, Funding acquisition, Formal analysis, Conceptualization.

## Declaration of competing interest

A.B. is inventor of a patent application (*WO/2020/254083A1 - Lipid nanocapsules charged with incretin mimetics*).

## Data Availability

Data will be made available on request.
